# Synchronous caecal and sigmoid volvulus: a surprise twist

**DOI:** 10.1093/jscr/rjab413

**Published:** 2021-09-22

**Authors:** Enda Hannan, Esther Man Yu Lim, Gerard Feeney, Eoghan Condon

**Affiliations:** Department of Colorectal Surgery, University Hospital Limerick, Limerick, Ireland; Department of Colorectal Surgery, University Hospital Limerick, Limerick, Ireland; Department of Colorectal Surgery, University Hospital Limerick, Limerick, Ireland; Department of Colorectal Surgery, University Hospital Limerick, Limerick, Ireland

## Abstract

Traditionally, caecal volvulus (CV) and sigmoid volvulus (SV) have been thought of as largely separate clinical entities with distinct clinical features, radiological findings and treatment strategies. We present a rare case of synchronous CV and SV. To our knowledge, this represents only the ninth such case in the literature. This posed a diagnostic challenge as the seemingly textbook features of SV, such as the classical ‘coffee-bean’ sign on plain abdominal X-ray, masked the simultaneous occurrence of CV which only became apparent after the patient continued to deteriorate despite the successful endoscopic decompression of the SV. The diagnosis of SV should be made cautiously, with a period of close clinical observation post-intervention and a low threshold for re-evaluation should symptoms persist or recur to ensure accurate diagnosis.

## INTRODUCTION

Volvulus is the axial rotation of a portion of the gastrointestinal tract along its supporting mesentery [1]. This most frequently occurs in the colon, resulting in bowel obstruction [1, 2]. This can lead to bowel necrosis, perforation and mortality [2]. For this reason, timely intervention is critical. The most common locations for colonic volvulus are the sigmoid colon (75%) and the caecum (22%) [3]. Typically, these are separate entities with distinct features and treatment strategies. Sigmoid volvulus (SV) is often seen in elderly, institutionalized patients with a history of chronic constipation [2, 3]. It is characterized by the ‘coffee-bean’ appearance on abdominal films and can be treated by endoscopic decompression in the acute phase provided that the bowel remains viable [2, 3]. This differs from caecal volvulus (CV) which is often seen in a younger demographic and almost always requires surgical resection [2].

The simultaneous occurrence of CV and SV is an exceedingly rare clinical entity, with less than 10 cases reported in the literature to date [1–5]. For this reason, this diagnosis can easily be missed and a potentially fatal delay to surgery can occur. In this report, we describe a rare case of synchronous CV and SV.

## CASE REPORT

**Figure 1:**
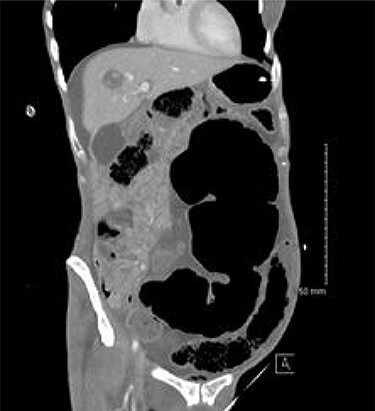
CT appearance suggestive of SV with collapsed right colon and no appreciable CV.

A 54-year-old female presented to the emergency department with a 2-day history of crampy abdominal pain, distension and absolute constipation. She had a history of chronic constipation but no other significant medical history. Her abdomen was diffusely tender and grossly distended on examination, with abdominal X-ray showing the ‘coffee bean’ appearance of SV. Computed tomography (CT) of the abdomen was performed which confirmed the presence of SV without evidence of ischaemia or perforation (Fig. 1). CV was not appreciated on this scan. The patient was brought to the endoscopy unit and underwent sigmoidoscopic decompression. Following this, the patient returned to the ward for observation.

**Figure 2:**
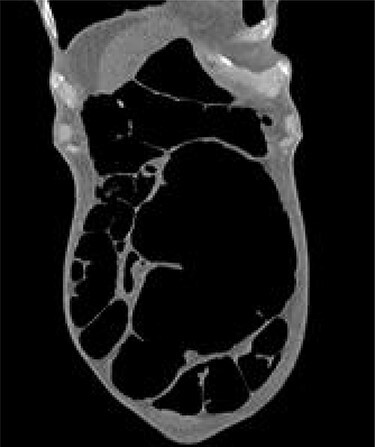
Grossly distended caecum on repeat CT scan.

The patient continued to experience abdominal pain with multiple episodes of vomiting post-procedure. A wide-bore nasogastric tube was placed which drained 850 ml of bilious fluid. New onset tachycardia was noted and physical examination raised concerns for peritonitis. A further abdominal CT was performed which showed that the SV had been successfully decompressed but also revealed the presence of CV with proximal small bowel dilatation (Fig. 2). There was pneumatosis in the wall of the caecum which was distended to 10 cm in diameter, raising concerns for ischaemia and impending perforation. The patient was promptly transferred to the operating theatre for exploratory laparotomy.

**Figure 3:**
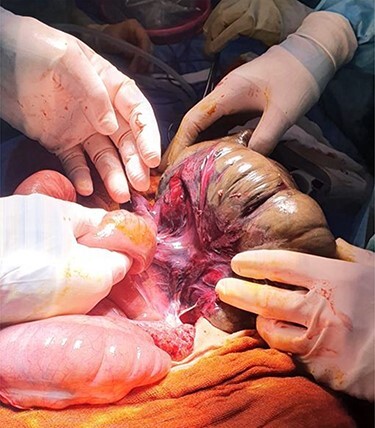
Intra-operative finding of CV with necrosis of the caecum. Long redundant sigmoid colon also evident.

A midline laparotomy was performed. A CV was found with necrosis of the caecum, which was highly mobile (Fig. 3). The SV remained decompressed but a long redundant sigmoid colon with a long mesentery was noted, posing risk for future recurrence. The decision was made to perform a total colectomy to treat both pathologies. An end ileostomy was created due to the decompensated status of the patient rendering anastomosis to be high-risk. The patient made a prompt recovery and was discharged on the sixth post-operative day. Histopathological analysis revealed no evidence of malignancy. She remains well at outpatient follow-up with the potential to undergo elective restoration of intestinal continuity in the future.

## DISCUSSION

A synchronous double volvulus of the colon affecting the caecum and sigmoid colon concurrently is an exceedingly rare condition. A detailed literature search revealed less than 10 previously reported cases [1–7] (Table 1). SV has long been considered to be a condition that has a classical presentation with easy-to-recognize clinical and radiological features [2]. However, in many of these reported cases, the seemingly textbook features of SV, such as the radiographic ‘coffee bean’ sign, masked the simultaneous occurrence of a CV. In many institutions, it would be standard practice to treat SV by endoscopic decompression based on X-ray appearances alone without first undergoing CT. This case highlights the importance to maintain a high index of clinical suspicion in such cases, with a low-threshold for operative management should the patient status deteriorate. Even with detailed cross-sectional imaging, it is also easy to miss the simultaneous occurrence of CV in the context of SV. In both our case and the report by Islam *et al.* [3], CV was not initially evident due to the mass effect exerted by the massively enlarged sigmoid which masked the presence of the underlying second pathology.

**Table 1:** Cases of synchronous CV and SV from the last decade

Author	Date	Journal	Location	Patient Age & Gender	Comorbidities	Diagnosis	Surgical Intervention	Postoperative Complications	Mortality
Berg *et al.* [5]	2015	Ugeskr Laeger	Denmark	69 year old male	Parkinson’s Disease, Hypertension	Intra-operative	Laparotomy, total colectomy and end ileostomy	Nil	No
Islam *et al.* [3]	2016	BMJ Case Reports	Trinidad	80 year old male	Hypertension, Ischaemic stroke	Intra-operative	Laparotomy, total colectomy and ileorectal anastomosis	Anastomotic leakage requiring laparotomy and end ileostomy formation	No
Roy *et al.* [1]	2019	BMJ Case Reports	Australia	77 year old male	Endovascular aortic aneurysm repair, lung malignancy	Intra-operative	Laparoscopic anterior resection, conversion to laparotomy following intra-operative diagnosis of CV, proceeded to total colectomy and end ileostomy	Bilateral pleural effusions, hospital acquired pneumonia	Yes
Butt *et al.* [4]	2021	Journal of Surgical Case Reports	United Kingdom	73 year old male	Myocardial infarction, hypertension, hypercholesterolaemia	Intra-operative	Laparotomy, subtotal colectomy and end ileostomy	Postoperative ileus managed conservatively	No
Butt *et al.* [4]	2021	Journal of Surgical Case Reports	United Kingdom	79 year old male	Hypertension, hypercholesterolaemia, benign prostatic hypertrophy	Intra-operative	Laparotomy, subtotal colectomy and end ileostomy	Adhesional small bowel obstruction managed conservatively	No

The underlying aetiology of colonic volvulus has long been debated, with a frequently proposed theory being that the stretching and lengthening of the mesentery occurs as a result of long-standing constipation [1]. It is also suggested that it may occur as the result of congenital variation, as it is well recognized by intestinal surgeons that some patients inherently have longer mesenteries with more mobile colon [1]. These theories would suggest that the common recognition of CV and SV as clinically distinct entities may be flawed, as both could arise from the same underlying aetiology, and thus, patients may be prone to developing both either on separate occasions or at the same presentation. In one previously reported case, the synchronous double volvulus was incidentally diagnosed intra-operatively by a vigilant surgeon after completion of a laparoscopic anterior resection for SV. This would suggest that, when performing surgery for SV or CV, it is a reasonable and safe modification of practice to outrule the presence of synchronous volvulus by thorough inspection of the colon intra-operatively [1].

Our case highlights a number of important learning points. The first is that the diagnosis of SV should be made cautiously, with a period of close clinical monitoring post-intervention being critical. Any subsequent deterioration or recurrence of symptoms should prompt thorough re-evaluation to ensure accurate diagnosis. The second is that CV and SV may not be truly distinct clinical entities as they have previously been described, potentially arising from the same underlying aetiology, and thus, patients that develop one may be at risk of the other. For this reason, it is worthwhile to outrule the presence of synchronous volvulus during surgery for what is initially suspected to be an isolated SV or CV. Finally, in the unusual case of concurrent SV and CV, the definitive surgical treatment is a total colectomy, with or without anastomosis depending on patient status, allowing for both pathologies to be treated. While there may be an argument to be made for organ-preserving techniques, such as sigmoidopexy or repeated endoscopic decompression, it would appear that the consensus from previously reported cases is that total or subtotal colectomy is the favoured surgical approach. This offers the advantage of eliminating the risk of recurrence while also allowing for histopathological analysis to outrule underlying malignancy. In many of these previously reported cases, patients had presented with symptoms on multiple occasions despite previous endoscopic management, highlighting the need for a definitive surgical solution.

## CONFLICT OF INTEREST STATEMENT

None to declare.

## FUNDING

No funding received.

## CONSENT

Obtained from the patient and documented in the medical notes.
